# Resistance and Security Index of Networks: Structural Information Perspective of Network Security

**DOI:** 10.1038/srep26810

**Published:** 2016-06-03

**Authors:** Angsheng Li, Qifu Hu, Jun Liu, Yicheng Pan

**Affiliations:** 1 State Key Laboratory of Computer Science, Institute of Software, Chinese Academy of Sciences, Beijing, P. R. China; 2 University of Chinese Academy of Sciences, Beijing, P. R. China

## Abstract

Recently, Li and Pan defined the metric of the *K*-dimensional structure entropy of a structured noisy dataset *G* to be the information that controls the formation of the *K*-dimensional structure 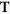 of *G* that is evolved by the rules, order and laws of *G*, excluding the random variations that occur in *G*. Here, we propose the notion of *resistance of networks* based on the one- and two-dimensional structural information of graphs. Given a graph *G*, we define the *resistance of G*, written 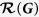, as the greatest overall number of bits required to determine the code of the module that is accessible via random walks with stationary distribution in *G*, from which the random walks cannot escape. We show that the resistance of networks follows the *resistance law of networks*, that is, for a network *G*, the resistance of *G* is 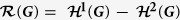, where 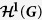 and 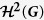 are the one- and two-dimensional structure entropies of *G*, respectively. Based on the resistance law, we define the *security index of a network G* to be the normalised resistance of *G*, that is, 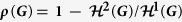. We show that the resistance and security index are both well-defined measures for the security of the networks.

An interesting recent discovery in network theory is that network topology is universal in nature, society, and industry^1^. In fact, the current highly connected world is assumed to be supported by numerous networking systems. Real-world networks are not only too important to fail, but also too complicated to understand.

Erdös-Rényi proposed the first model^2^,^3^ (hereafter referred to as the ER model) to capture complex systems based on the assumption that real systems are evolved randomly. The ER model explores the well-known small-diameter property of networks, that the diameter of a network of *n* nodes is *O*(log *n*); this property is the essence of the small-world phenomenon, and is the first general property of networks. The small-world phenomenon of networks is simply guaranteed by some randomness in the sense that, for any graph, if we add a small number of edges randomly and uniformly in the graph, the diameter of the new graph is small with high probability. However, real-world networks are not purely random. Barabási and Albert^4^ proposed a graph generator by introducing preferential attachment as an explicit mechanism; the model is thus called the preferential attachment (PA) model. Consequently, networks generated by the PA model naturally follow a power law. It has been shown that most real networks follow a power law; this is the second universal property of networks^1^.

Networks may fail due to different ways of attacks and different mechanisms of failure^5^,^6^,^7^,^8^,^9^. The first type is physical attack via removal of some nodes or edges. It has been shown that in scale-free networks generated by the preferential attachment (PA) model^4^, the overall network connectivity as measured by the sizes of the giant connected components and the diameters does not change significantly in response to random removal of a small fraction of nodes but is vulnerable to removal of a small fraction of high-degree nodes^9^,^10^,^11^. The second type is the cascading failure of attacks, which naturally appears in rumour spreading, disease spreading, voting, and advertising^5^,^6^,^12^. It has been shown that in scale-free networks generated by the PA model even a weakly virulent virus can spread^13^. This result explains a fundamental characteristic of the security of networks^8^.

For physical attacks or random errors from removal of nodes, it was shown that optimal networks capable of resisting both physical attacks and random errors have at most three degree values for all of the nodes of the networks^14^, and that networks that have optimal robustness to both high-degree node attacks and random errors have a bimodal degree distribution^15^. These results are all related to security or robustness in the face of physical attacks or random errors. Notably, the graphs that are characterized as secure or robust are far from real graphs; they have only two or three choices of degree for the nodes, which never occurs in real networks. Callaway, Newman, Strogatz and Watts^16^ studied robustness and fragility based on the notion of percolation on random graphs, and Cohen, Erez, ben-Avraham and Havlin^10^,^17^ studied the resilience of networks to random breakdowns and intentional attack.

To enhance the robustness of networks against the spread of biological viruses, the acquaintance immunization strategy was proposed^18^. This strategy involves immunization of random acquaintances of randomly chosen nodes. More recently, a security-enhancing algorithm that randomly swaps two edges for a number of pairs of edges was proposed^19^.

Real-world networks are highly connected and naturally evolving, and information can spread in them easily and quickly. One of the main features of networks in the current highly connected world is that the failure of a few nodes of a network may generate cascading failure throughout the network. It is possible that a small number of attacks or even random errors may generate global network failure. For instance, the failure of a few US commercial banks was the beginning of the 2008 global financial crisis, which eventually spread throughout the world. Increasingly many economic activities are based on the Internet; for instance, the rapidly growing financial and business networks in China are of vital importance, and their security must be guaranteed.

Li *et al*.^20^ proposed a security model based on the idea of the Art of War^21^. It has been shown that with the appropriate parameters, networks generated by the security model are provably secure against any small-scale virus attack (Li, and Pan, A theory of network security: Principles of natural selection and combinatorics, Internet Mathematics, to appear).

However, some fundamental questions are not addressed by Li and Pan: what are the measures of the security of a network? What is the principle that guarantees the security of the networks generated by the security model? In addition, we don’t know why networks generated with the PA model are so vulnerable to intentional attacks for all failure mechanisms, including the cascading model of virus attacks, physical attacks and biological virus attacks.

The above questions are closely related to the challenge posed by Shannon in 1953^22^, who found that his definition of information fails to support communication network analysis; he proposed the question of whether there is a metric to define the information that is embedded in physical structures such as networks. In 2003, Brooks^23^ suggested the missing theory of structural information as the first of three half-century-old challenges in computer science.

Li and Pan (Li, A. and Pan, Y. Structural Information and Dynamical Complexity of Networks, IEEE Transactions on Information Theory, to appear) proposed the metric of *K*-dimensional structure entropy of graphs to measure the complexity of the interactions, communications and operations in graphs. Equally important, the *K*-dimensional structure entropy of a network *G* (a structured noisy dataset) provides a principle that makes it possible to distinguish the structure of *G* that is formed by the rules, order and laws of *G* from the structure of *G* that is formed by random variations. This provides a foundation for data science and knowledge discovery based on noisy data that are both structured and unstructured. Li, Li and Pan^24^ have shown that two-dimensional structure entropy minimisation can be used to discover natural communities in social and biological networks. Li *et al*.^25^ proposed a homophyly/kinship model based on Darwin’s idea of natural selection and showed that structure entropy minimisation reflects the principle of natural selection in networks that are naturally evolving. This idea suggests the natural thesis that structure entropy minimisation is the principle of natural selection in nature and society, leading to new mathematics in general science. Li, Yin and Pan^26^ have shown that two- and three-dimensional structure entropy minimisation is successful at defining cancer cell types and subtypes.

Here, we propose the notion of the resistance of a network based on the notion of structural information to quantitatively measure the force of the network to resist cascading failures caused by intentional virus attacks.

We show that the resistance of a network does measure the dynamics of the network resisting cascading failure of virus attacks on the network, and that resistance maximisation is a useful principle for security of networks. We find the *local resistance law of networks*, that is, for a connected network *G* = (*V*, *E*) and a partition 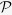 of *G*, the resistance of *G* given by 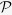 is 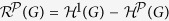, where 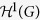 is the one-dimensional structure entropy of *G*, and 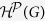 is the structure entropy of *G* given by partition 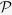. We also find the *global resistance law of networks*, that is, for a connected graph *G*, the resistance of *G* is 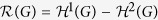, where 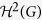 is the two-dimensional structure entropy of *G*. The local resistance law of networks allows us to secure a network *G* by finding the partition 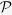 such that the resistance of *G* given by 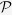 is maximised.

We show that for the PA model, the resistance and security index of a network are both robust to random variations and exponentially decrease as *d* increases. We demonstrate that for a network of the security model with appropriate choices for the affinity exponent, the resistance and security index are both robust to random variations in the model and are invariant to *d* > 1, and that for a network model, including the PA model, the security model, and dynamical random model (in the case of the security model with affinity exponent *a* = 0), the security of the networks against cascading failure caused by a small-scale virus attack is measured by both the resistance and security index of the networks with a slight perturbation by the random variations in the models; finally, we show that for real-world networks, the security of the networks against cascading failure caused by a small-scale virus attack is truthfully characterised by both the resistance and security index of the network. The results demonstrate that both the resistance and security index are well-defined measures of security against intentional virus attacks.

Our theory demonstrates that the structural information proposed by Li and Pan does support network analysis, as anticipated by Shannon in 1953. The research presented in this study is the first step toward a foundation for engineering networks, including communication networks, computer networks and computing systems.

## The Challenges

Shannon^22^ proposed the question of whether there is an information theory that supports analysis of communication networks and that generates optimal communication systems. Since the publication of Shannon’s study 60 years ago, there has been no substantial progress reharding these questions. As Brooks^23^ commented, “We have no theory, however, that gives us a metric for the information embedded in structure, especially physical structure” and “I consider this missing metric to be the most fundamental gap in the theoretical underpinnings of information science and of computer science”.

As Shannon^22^ noted, his definition of information fails to support network analysis. The reason is as follows:

Given a network *G* = (*V*, *E*), to compute the Shannon information of *G*, we have to first define a distribution *p* = (*p*_1_, *p*_2_, ···, *p*_*l*_) from *G*, and then compute the Shannon information of *p*, i.e., 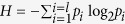 as the information of *G*. However, the Shannon information *H* is a number that tells us little regarding the properties of *G*. In the procedure above, regardless of the *G* distribution used, we lose information regarding the structure of *G*, which is certainly the most important property of *G*. Therefore, the Shannon information is defined as a number associated with a distribution extracted from *G*, and the Shannon number fails to preserve most properties of *G*.

The challenge posed by Shannon is so fundamental for many reasons, including the following:

1. Given a communication network *G*, there are usually a number of interactions, communications and operations that occur simultaneously within the network. How can we guarantee that the network *G* always works properly?
2. Suppose that *G* evolves naturally in nature and society. There are certain rules, regulations and laws that control the evolution of *G*, and simultaneously, there are random variations in the evolution of *G*. How can we distinguish between the part of *G* that is formed by rules, regulations and laws and the part of *G* that is formed by random variations? If this problem were solved, we would be able to distinguish natural selection from random variations in the evolution of nature and society, and we would thus be able to extract true knowledge from noisy data.
3. Given a network *G*, there are viruses that randomly walk in *G*. How can we catch the viruses?
4. What are the principles behind the security of networks?

Structural information theory (Li, A. and Pan, Y. Structural Information and Dynamical Complexity of Networks, IEEE Transactions on Information Theory, to appear) solved problems 1), 2) and 3) above. Here we will solve 4).

## Structural Information

To establish our theory, we introduce the closely related one- and two-dimensional structure entropies of graphs by proposed Li and Pan.

### One-dimensional structure entropy: positioning entropy

Let *G* = (*V*, *E*) be a connected graph with *n* nodes and *m* edges. For each node *i* ∈ {1, 2, ···, *n*}, let *d*_*i*_ be the degree of *i* in *G*, and let *p*_*i*_ = *d*_*i*_/2*m*. Then, the vector *p* = (*p*_1_, *p*_2_, ···, *p*_*n*_) is the stationary distribution of a random walk in *G*.

We define the *one-dimensional structure entropy of G* or the *positioning entropy of G* as follows:


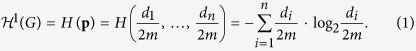



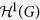
 is the amount of information required to determine the code of the node that is accessible from the random walk with the stationary distribution in *G*. It is a dynamic notion regarding random walks that differs from the Shannon entropy to determine the code of the node by random selection among the nodes of the graph.

Remarks: (i) The definition of 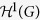 can be easily extended to edge-weighted graphs, in which case the degree of a node is defined as the sum of the weights of all of the edges connected to the node. (ii) If the graph *G* is disconnected, the one-dimensional structure entropy of *G* is the weighted average of the one-dimensional structure entropies of all of the connected components of *G*. (iii) If *G* consists of a single isolated node, the one-dimensional structure entropy of *G* is 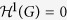, because no random walk in *G* is possible.

### Two-dimensional structure entropy: Structure entropy

Given a connected graph *G* = (*V*, *E*), suppose that 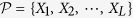 is a partition of *V*. By using the partition 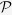, we encode a node *v* ∈ *V* by a pair (*i*, *j*) such that *i* is the code of node *v* in the module 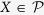 that contains *v*, and *j* is the code of the module 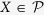 that contains *v* in *G*.

We define the *two-dimensional structure entropy of G given by* 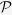, which is also referred to as the *structure entropy of G by* 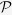, as follows:


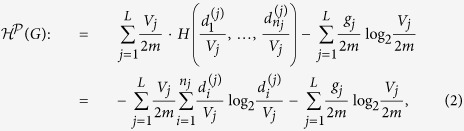


where *L* is the number of modules in partition 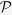, *n*_*j*_ is the number of nodes in module *X*_*j*_, 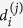 is the degree of the *i*-th node in *X*_*j*_, *V*_*j*_ is the volume of module *X*_*j*_ (i.e., the sum of the degrees of all the nodes in *X*_*j*_), *g*_*j*_ is the number of edges with exactly one endpoint in module *j*, and *m* is the number of edges in *G*, and 2*m* is the volume of *G*.


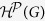
 consists of two parts: the first part is the information of the node in its own module, and the second part is the information of the module that is accessible from random walks from nodes outside the module. The intuition of the definition is as follows: the first part corresponds to the local number of a phone call, and the second part corresponds to the area codes for a distant call. In a phone call, one always needs a local phone number, but one needs an area code only for distant calls. A phone call within the same area only requires the local phone number. This feature is reflected in the second part of the definition in the sense that we need to determine the code of the module only if a random walk arrives at the module from nodes outside the module.

According to the definition, 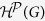 is the average number of bits required to determine the code (*i*, *j*) of the node that is accessible from random walks with stationary distribution in *G*, where *i* is the code of the node in its own community and *j* is the code of the community of the accessible node.

Suppose that 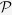 is an optimal partition of *G*. Then, the structure entropy of *G* given by 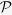 is minimised. In this case, by using the partition 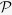, locating the viruses that randomly walk in *G* is easy. However, how can we compute the optimal partition 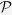
? For this, we define the *two-dimensional structure entropy*, which is also referred to as the *structural information* of networks.

Given a connected graph *G*, define the *two-dimensional structure entropy of G* (also known as the *structure entropy of G*) as follows:


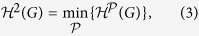


where 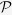 runs over all of the partitions of *G*.

According to the definition presented in Equation (3), the following hold:

1. For a connected graph *G*, the two-dimensional structure entropy of *G* is the least overall number of bits needed to define the two-dimensional code of the node that is accessible from the random walk with stationary distribution in *G*.
2. The optimal partition 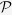 of *G* is controlled and achieved by the two-dimensional structure entropy 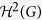 of *G*.
3. The two-dimensional structure entropy 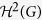 of *G* is still a number. However, the number 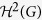 provides a principle for us to define the optimal partition 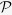 of *G*.
4. The optimal partition 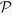 of *G* is the two-dimensional structure, i.e., the community structure of *G* that minimises the non-determinism or uncertainty of random walks in *G*. Thus 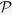 preserves the structure of *G* against random variations. Therefore, most properties of *G* that are formed by the rules, regulations and laws of *G* are preserved in 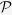. Suppose that 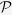 is a partition of the vertices of *G* such that 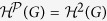. We then say that *G* has two-dimensional structure entropy 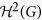 with an accompanying two-dimensional structure 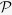. Clearly, if 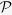 is an accompanying structure of *G* with two-dimensional structure entropy 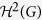, the knowledge of the rules, regulations and laws of *G* can be extracted from 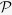. This approach provides a foundation for knowledge discovery from the noisy network *G*.
5. In mathematics, the notion 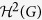 provides a new metric to characterise graphs, including graphs of classic data and big data in general. Such characterisations reveal us the complexity of the dynamical interactions in the graphs.
6. In algorithmic theory, the computation of 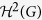 is a new algorithmic problem, for which the time and space complexity and the hardness of the problem are interesting open questions.
7. In practice, there are many methods to approximate the value of 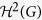:

- Start with the trivial partition 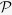 such that each module contains only one node,
- Introduce reasonable operators for merging two modules in 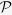,
- Introduce reasonable operators for splitting a module in 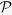 into two submodules, and
- Greedily apply one of the operators above iteratively such that the reduction of the two-dimensional structure entropies of the two corresponding partitions is maximised among all the operators applicable in the current step. This procedure yields an approximate value for 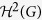 with an accompanying partition 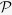.

The approach above provides abundant opportunity for improved approximate algorithms for computing 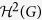.

We have shown that the algorithm of the approach using only the naive merging operator in (ii) above is already remarkably better than the existing algorithms in detecting natural communities in social networks and biological networks and for defining cancer cell types and subtypes^24^,^25^,^26^.

Define the *normalised structure entropy of G* as follows:


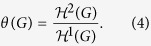


For a connected network *G*, the normalised structure entropy of *G* measures the compression ratio of the network *G*.

Clearly, the two-dimensional structure entropy of graphs can be naturally extended to high-dimensional cases, in which case a node is encoded by a *K*-dimensional vector of codes. To define the high-dimensional structure entropy of a graph *G*, we introduce the notion of a partitioning tree 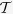, define the structure entropy of *G* given by the partitioning tree 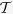 and define the *K*-dimensional structure entropy of *G* to be the least structure entropy of *G* given by the *K*-level partitioning trees among all the *K*-level partitioning trees of *G*. We say that a height *K* partitioning tree 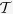 of *G* is a knowledge tree of *G*, if 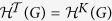, where 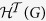 is the structural information of *G* given by 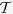, and 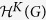 is the *K*-dimensional structure entropy of *G*. The notion of a knowledge tree of networks provides a foundation for knowledge discovery. As an example, Li, Yin and Pan^26^ have shown that one-dimensional structure entropy minimisation is a useful principle for constructing networks for unstructured data and that the two- and three-dimensional knowledge trees can be used to determine the cell types and subtypes for a number of cancers.

The Li-Pan structural information and the Shannon information are essentially different. The the notable differences between the two metrics are:

- The Shannon information performs a de-structuring of a network *G* and yields the Shannon entropy of *G*, which tells us the degree of uncertainty in *G*. Shannon entropy “kills” *G* by cutting off the connections in *G*.
- The *K*-dimensional structure entropy of *G* is the information of *G* that determines and decodes the accompanying structure 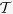 (a partitioning tree) of *G* such that 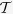 is obtained from *G* by excluding the maximum amount of the non-determinism or uncertainty that have occurred in *G*. The structural information of *G* distinguishes between the part of *G* generated by order and the part of *G* caused by noises and random variations.

## Resistance of Networks

Given a network *G* = (*V*, *E*), assume that a virus randomly spreads in *G*. What is the condition under which the virus cannot spread throughout the network? Suppose that there is a partition 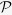 of *G* such that a random walk with stationary distribution in *G* easily goes to a small module *X* of 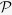, after which it is difficult for the random walk to escape from the module *X*. Based on the assumptions regarding 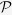 and *G*, a virus from any node of *G* very likely goes to a small module *X* of 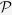, after which it is difficult for the virus to infect nodes outside of *X*. This intuition leads us to define the *resistance of G given by a partition* 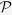.

Given a connected network *G* = (*V*, *E*), let 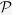 be a partition of *G*. We define the *resistance of G given by* 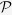 as follows:


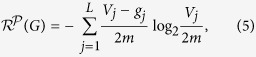


where *V*_*j*_ is the volume of the *j*-th module *X*_*j*_ of 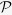, *g*_*j*_ is the number of edges from *X*_*j*_ to nodes outside *X*_*j*_, and *m* is the number of edges in *G*.

In Equation (5), for the *j*-th term 
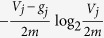
, 
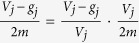
 is the probability that a random walk goes to the *j*-th module *X*_*j*_ and fails to escape from the *j*-th module *X*_*j*_, and 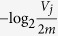 is the number of bits to determine the code of the *j*-th module in *G*. Therefore, 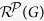 is the average number of bits required to determine the code of the randomly accessible module that hinders the random walk from spreading from the nodes of the module to nodes outside the module. Intuitively, 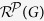 is the resistance of *G* given by 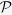.

Now, we are ready to define the *resistance of a graph G* as follows:


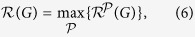


where 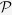 runs over all partitions of *G*.

According to the definition, 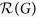 is the maximum overall number of bits required to determine the code of the module of *G* that is accessible from random walk and from which random walk cannot escape. Intuitively, 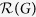 is the force of *G* to resist cascading failure caused by intentional virus attacks on *G*.

As in the case of the two-dimensional structure entropy, computation of the exact value 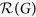 seems difficult because it is defined over all partitions of *G*. However, approximate solutions for 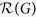 can be computed greedily using the same approach as for 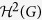. Therefore, we have that the number 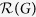 provides us with a principle for finding the partition 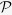 of *G* that protects network *G* from cascading failure caused by virus attacks. Thus, the metric 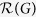 not only quantifies the force of the network to resist virus attacks but also provides us with a two-dimensional structure 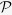 of *G* that protects and controls the network *G*. The latter result means that the notion of the resistance of networks provides us with a principle for both security and control of networks.

## Resistance Law of Networks

Let *G* = (*V*, *E*) be a connected graph. Suppose that 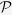 is a partition of *V* with the notations the same as those in the definitions of 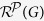, 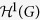 and 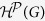. Then the positioning entropy of *G*, 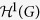, and the resistance and structure entropy of *G* by 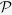, i.e., 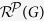 and 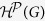, have the following properties:

(1) (Additivity of 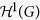) The positioning entropy of *G* satisfies:


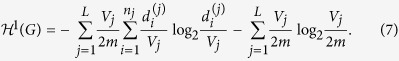


(2) (Local resistance law of networks)


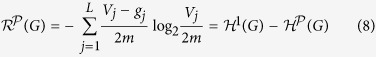


Given a graph *G* = (*V*, *E*) and a set *X* ⊂ *V*, we define the conductance of *X* in *G* by


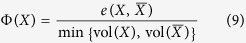


where 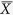 is the complement of *X*, 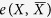 is the number of edges between *X* and 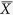, vol(*Y*) is the volume of *Y* in *G*, for *Y* ⊂ *V*.

(3) Assume that for each *j*, *V*_*j*_ ≤ *m*, for *m* = |*E*|. Then


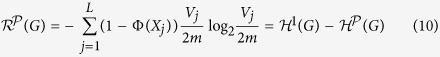


where Φ(*X*_*j*_) is the conductance of *X*_*j*_ in *G*.

We prove the properties in (1)–(3) above as follows. By the definition in Equations (1) and (2), for the partition 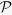 of *V*,


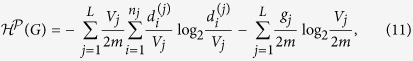


and


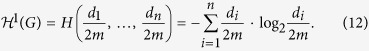


By the additivity of the entropy function, for the partition 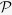,


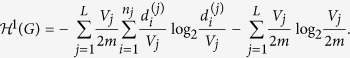


(1) follows.

The resistance of *G* by 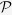 is



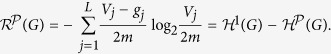



(2) follows.

For every *j* ∈ {1, 2, ···, *L*}, let Φ_*j*_ be the conductance of *X*_*j*_ in *G*, i.e., Φ_*j*_ = Φ(*X*_*j*_). Then, for every *j* ∈ {1, 2, ···, *L*}, if *V*_*j*_ ≤ |*E*|, then 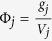 .Assume that for each *j*, *V*_*j*_ ≤ *m*, for *m* = |*E*|. By (2), we have



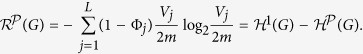



(3) follows.

This establishes the resistance principle of networks given by partitions.

By the definition of the resistance of *G*, the local resistance law in (2) above and the definition of the two-dimensional structure entropy, we have the following:

*Global resistance law of networks*: for a network *G*, we have


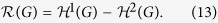


According to the global resistance law, we define the *security index of G* to be the normalised resistance of *G* as follows:


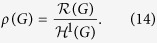


Based on the global resistance law given by Equation (13) and the definition of a security index given by Equation (14) , the security index of *G* is


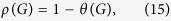


where *θ*(*G*) is the normalised structure entropy of *G*.

## High Resistance Guarantees the Security of Networks

Intuitively, given a graph *G*, if the resistance 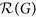 of *G* is high, there is a partition 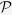 of the vertices of *G* such that 
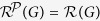
 is high. This property implies that i) and ii) below hold.

- Most modules 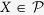 are small.
- It is hard for random walks to go from a module 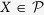 to a different module 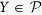.

We argue as follows. By definition,


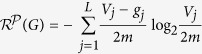


For (i). Towards a contradiction, suppose that (i) fails to hold. Then, there are many large 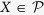. Let *X* = *X*_*j*_ be such a module. Then, 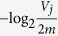 is small.

If there are many large modules *X*_*j*_ in 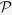, 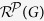 cannot be large.

For (ii). Suppose to the contrary that there are many modules *X*_*j*_ such that the number of edges from *X*_*j*_ to nodes outside *X*_*j*_ is large. For those *j*’s, 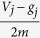 are small.

If there are many such modules *X*_*j*_ in 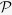, 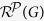 cannot be large.

(i) and (ii) ensure that random walks in *G* easily arrive at some small module *X* in 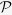, after which it is hard to escape. Due to the global maximality of 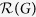, if the resistance 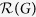 is large, random walks of a virus from any starting node can infect only a module *X* that is small. Furthermore, a small number of viruses from any starting points can infect at most a small number of small modules *X* in 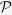.

In this report, we define the security of a network *G* as follows. Given a network *G* = (*V*, *E*), a natural number *k* and a small number 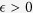, we say that *G* is 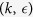-secure, if:

With probability ≈1, for any set *S* ⊂ *V*, if the size of *S* is ≤*k*, then virus attacks on all of the nodes in *S* infect at most 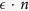 nodes in *V* in a cascading failure model.

The cascading failure model works with random thresholds, for which the details are referred to the Methods section.

*Remark*: We assume that a virus spreads and infects in a random manner. However, the attacks are selected by clever people, and thus security must be able to forestall all possible attacks.

In our definition above, the security is measured by *k* and 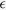, the security of *G* requires that *k* is appropriately large, and 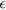 is small. Theoretically, we allow *k* to be 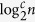 for any constant *c* > 0, if *n* is sufficiently large, and 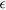 approaches 0 if *n* goes to infinity^27^.

We will show that the resistance and security index characterise the security of networks defined above. Particularly, we establish the following *security principle of networks*:

- Given a network *G*, the resistance 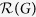 of *G* and the security index *ρ*(*G*), characterise the security of *G* against cascading failure caused by intentional virus attacks on *G*.
- Given a model of networks, in most cases, both the resistances and security indices of networks of the same type are robust to random variations in the model.
- For a model of networks, the security of the networks of the same type of the model is always sensitive to random variations in the model.
- For a model of networks, the security of the networks of the model is characterised by the resistances and security indices of the networks with perturbations of random variations in the model.

## The PA Model

The networks generated by the PA model^4^ have already been shown to be fragile in the face of intentional attacks based on various failure mechanisms, including physical attacks, virus attacks, cascading failure and the SIR model^5^,^6^,^7^,^8^,^9^,^10^,^11^,^12^,^13^.

Here we investigate the resistances and security indices of the networks of the PA model, from which we now know why the networks of the PA model are vulnerable to intentional attacks using various mechanisms of failure.

**Figure 1:**
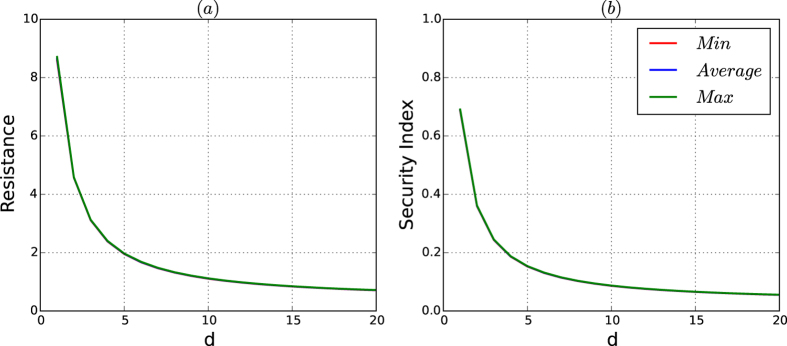
Resistances and security indices of the networks of the PA model. The number of nodes is 10,000. (**a**) Depicts the minimum, average and maximum of the resistances of the networks, and (**b**) depicts the minimum, average and maximum of the security indices of the networks. The minimum, average and maximum of the networks for each type are taken over 200 networks. For each network, the resistance and security index of the network are computed by using the resistance maximisation algorithm 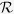.

In Fig. 1(a,b), we depict the maximum, average and minimum of the resistances and security indices, respectively, of networks composed of nodes *n* = 10,000 nodes generated by the PA model. In this experiment, for each type with different *d*’s, we generate 200 networks. For each network, we compute the resistance and security index of the network by the partition found by our resistance maximisation algorithm 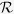, which is described in the Methods section. The minimum, average and maximum resistance and security index for each type are computed over all of the 200 networks of the type.

From Fig. 1, we observe the following results:

1. For resistance, according to Fig. 1(a), we have:

- (Robustness) For every type *d*, the curves of the average, minimum and maximum of the resistance of the 200 networks of the PA model given by the resistance maximisation algorithm 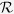 coincide. This means that the resistance of the networks of the PA model is robust to random variations in different generations of the model and is determined by the type (*n*, *d*) of the networks.
- The resistance of the networks decreases dramatically as *d* increases from 1 to 5 and decreases slowly as *d* increases from 5.
- (High resistance) The resistances of the networks are non-trivially high only if *d* is trivially small or less than 5.
- (Low resistance) For *d* ≥ 5, the resistances of the networks of the PA model are trivially small, say, less than 2.
- (Exponentially decreasing property) The curve of the resistances of the networks of the PA model in Fig. 1(a) can be approximated by a function of the following form:

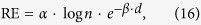


for some constant *α* and *β*, where *n* is the number of nodes and *d* is the average number of edges of the network.

1. For the security indices, from Fig. 1(b), we have the following:

- (Robustness of the security index) The curves of the minimum, average and maximum of the security indices of the networks of the PA model are similar to those of the corresponding resistances of the networks in Fig. 1(a).
- (Exponentially decreasing property) The coincident curve of the minimum, average and maximum of the security indices can be approximately modelled by a function of the following form:

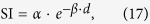


where *d* is the number of average edges, *α* and *β* are approximately equal to the corresponding constants in Equation (16).

The results in (1) and (2) demonstrate that the notion of the resistance, the security index and the two-dimensional structure entropy are robust to the random variations in the PA model and that both the resistance and security index exponentially decrease as *d* increases. We will show that the resistance and security index given by Equations (16) and (17), respectively, characterise the security of the networks of the PA model together with a perturbation from random variations.

**Figure 2:**
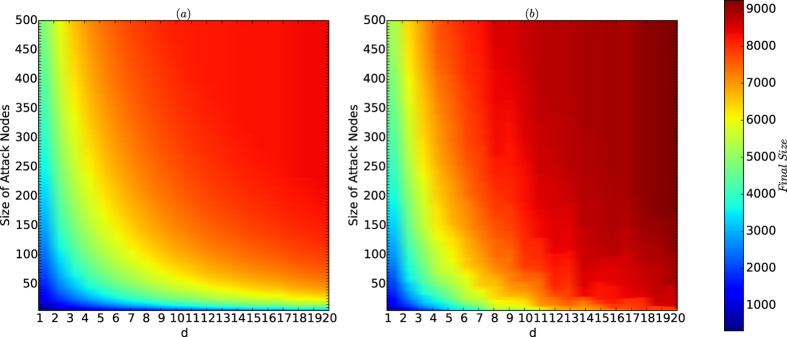
Colour codes of *I*_avg_ and *I*_max_ for the networks of the PA model. (**a**) Depicts the colour codes for the average sizes of cascading failure sets, and (**b**) depicts the maximum sizes of the cascading failure nodes. In both (**a,b**), the horizontal line represents the parameter *d*, and the vertical line represents the size of attacks. In this experiment, the number of nodes *n* = 10,000, and *N* = *M* = 200. The parameter *d* ranges from 1 to 20, and the size *k* of viruses ranges from 1 to 500 with unit 1. The most influential *k* nodes are selected by the algorithm CLC, which is currently the best algorithm for finding the most influential nodes in networks.

Figure 2(a,b) depict the colour codes of the average and maximum of the sizes, respectively, of the cascading failures of virus attacks on the networks of the PA model. All the networks have size *n* = 10,000. *d* ranges from 1 to 20. For each type, we generate 200 networks. For each of the 200 networks and for each size *k* of viruses, we implement virus attacks 200. For each attack, we define the threshold *ϕ*(*v*) for every node *v* of the network to be a random number, and we attack the most influential *k* nodes found by the current best combinatorial local centrality (CLC) strategy; see the Methods section. The average and maximum sizes for each type and each size *k* are computed for all 200 times of attacks for each of the 200 networks.

According to Fig. 2, we have the following results:

1. For both average and maximum cases in Fig. 2(a,b), there are golden belts that are similar to the resistance curve in Fig. 1(a) and the security index curve in Fig. 1(b) and that determine the secure areas of the networks.
2. The secure areas for the average and maximum of the sizes of cascading failure in Fig. 2(a,b), respectively, are slightly different, meaning that the security of the networks of the PA model is sensitive to random variations in the model (the variations occurred in different generations of the same type, i.e., the same *n* and the same *d*). However, as we have seen from Fig. 1, the resistance and security index of the networks of the PA model are robust to random variations in the model. Therefore, the security of the networks of the PA model is characterised by the resistances and security indices of the networks with perturbations due to random variations in the model.
3. The security of the networks of the PA model exponentially decreases as *d* increases, as characterised by the security indices of the networks of the PA model.
4. The result demonstrates that the resistances and the security indices of the networks characterise the security of the networks, with slight perturbations due to random variations in the model, and that the security of the networks of the PA model is determined by a function of the form similar to that in Equations (16) and (17).

## Security Model

Li *et al*.^20^ introduced the security model of networks. The security model proceeds as follows:

Given an *affinity exponent a* ≥ 0 and a natural number *d*,

1. Let *G*_*d*_ be an initial *d*-regular graph such that each node has a distinct *colour* and is called a *seed*. For each step *i* > *d*, let *G*_*i*−1_ be the graph constructed at the end of step *i* − 1, and *p*_*i*_ = 1/(log*i*)^*a*^.
2. At step *i*, we create a new node, *v*.
3. With probability *p*_*i*_, *v* chooses a new colour, in which case,
  - we call *v* a seed,
  - (PA) create an edge (*v*, *u*), where *u* is chosen with a probability proportional to the degrees of nodes in *G*_*i*−1_, and
  - (randomness) create *d* − 1 edges (*v*, *u*_*j*_), where each *u*_*j*_ is chosen randomly and uniformly among all seed nodes in *G*_*i*−1_.
4. Otherwise, *v* chooses an old colour, in which case
  - (randomness) *v* uniformly and randomly chooses an old colour as its own colour and
  - (homophyly and PA) create *d* edges (*v*, *u*_*j*_), where *u*_*j*_ is chosen with a probability proportional to the degrees of all nodes of the same colour as that of *v* in *G*_*i*−1_.

The model is dynamic; the maximum number of *i* can be an arbitrarily given natural number *n*. For fixed affinity exponent *a*, average number of edges *d* and natural number *n*, we use 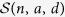 to denote the set of networks generated by the security model with number of nodes *n*, average number of edges *d* and affinity exponent *a*.

The model simulates the growth of the real-world Internet in the following sense:

1. When a new individual *v*, a computer or a person, is born, *v* has its own characteristics, playing either a local role in an existing community or a global role that leads to a new community. For a network of the security model, we say that the set of all nodes of the same colour for a fixed colour is a natural community or simply a community.
2. If an individual *v* plays a local role, it joins some existing community randomly, in which it links to existing nodes of the randomly chosen community by following the rich-get-richer mechanism.
3. If an individual *v* plays a global role, it creates links by both the preferential attachment mechanism and random selection of seed nodes (or king nodes). (2) and (3) are very similar to the formation of social groups in nature, such as formation of the colonies of honey bees. One of our original ideas for the security model is based on the idea that the species that survived the evolutionary process in nature may have mechanisms to protect themselves, based on which the mechanisms of the security of networks may be derived.
4. The affinity exponent *a* reflects the degree to which an individual likes to join an existing community. If *a* is small, an individual is more likely to be a king node that leads a community, whereas if *a* is large, an individual is more likely to join an existing community.

It can be shown that the size of a community is bounded by 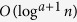 for a network in 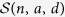.

**Figure 3:**
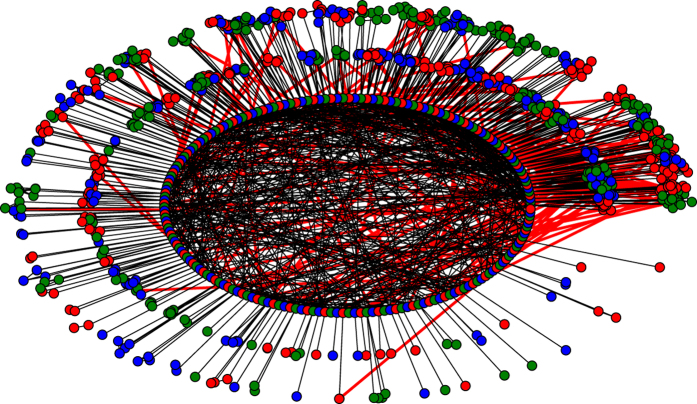
A network of the security model with *n* = 1,000, *a* = 1 and *d* = 4. Seed nodes are red, and non-seed nodes are blue.

In Fig. 3, we depict a network from the security model with *n* = 1,000, *a* = 0.8 and *d* = 4. In Fig. 3, the innermost circle represents the seed nodes, and the two outer circles represent the natural communities such that each community is depicted as the module sharing the same colour with its corresponding seed node.

We analyse the security of the networks of the security model as follows.

According to Fig. 3, the graph *G* generated by the security model satisfies the following properties:

- A natural community, that is, the maximal set of nodes of the same colour, is small, with one seed node, such that the number of communities is large.
- The degree of a seed node is largely contributed by nodes of its own community.
- A seed node links to at most one non-seed node outside its own community. Thus, there are only a small number of edges, i.e., the edges from the innermost circle to the two outer circles that are colored red and those from seed nodes in the innermost circle to the nodes in the two outer circles that are not in their own communities.
- The links among the seed nodes, i.e., the edges within the most inner circle and colored black, are randomly and evenly distributed.

(i) ensures that even if a node *x* in a community *X* infects the whole community *X*, an infection of the graph *G* is still a local infection. (ii) ensures that for a seed node *x*_0_ of community *X*, if none of the nodes in *X* has been infected, it is hard for *x*_0_ to be infected by its neighbours outside *x*_0_’s own community *X*. (iii) ensures that the infection of the seed node *x*_0_ of a community *X* may cause at most one non-seed node *y* outside *X* to be infected. (ii) and (iii) together ensure that the infections among different communities started from an infected seed are linearly increasing and that the length of the infection chain is short, *O*(log *n*) in theory. Therefore, attack from a small number of viruses may infect only a small number of chains of communities such that each of the chains is short. Again, by (i), the total number of nodes infected must be small compared to the size *n* of *G*. (iv) ensures that it is hard to select a small number of nodes for the virus attacks.

Mathematical proofs of the security theorems are given in (Li and Pan, A Theory of Network Security: Principles of Natural Selection and Combinatorics, Internet Mathematics, to appear).

This theoretical result shows that the networks are provably secure against intentional virus attacks. However, the theoretical result cannot be applied to practice directly because there are hidden constants in the *o*- and *O*-notations and the theoretical result holds only for sufficiently large *n*. In practice, *n* is bounded by a constant, and the values in the *o*- and *O*-notations are essential.

Here, we study the resistances and security indices of the networks of the security model, from which we learn not only the provable security result but also why the networks are secure.

## Resistances and Security Indices of the Networks of the Security Model

We investigate the resistances and security indices of the networks given by the resistance maximisation algorithm 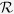 for the networks generated by the security model and the security of the networks against cascading failure of attacks.

For all experiments for the security model, the number *n* of nodes is fixed to *n* = 10,000. A type is determined by a triple (*n*, *a*, *d*). For each type, we generate 200 networks.

For the experiments regarding the resistance and the security index, we do the following: for each of the 200 networks of a fixed type, we compute the resistance and security index of the network based on the partition found by the resistance maximisation algorithm 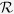. For each type, we compute the minimum, average and maximum of the resistances and security indices of the 200 networks.

For the security experiments, we implement the following: for each of the 200 networks of a given type and for each size *k* of viruses, we implement an attack 200 times. For each of the 200 attacks, we define the threshold to be a random number for every node of the network and select the most influential *k* nodes as the nodes to be infected by a virus. We compute the cascading failure set of the virus attacks on the selected *k* nodes. For each type and each size *k* of the viruses, we compute the average and maximum sizes of the cascading failure sets over all the attacks of the networks for the type with *k* viruses.

### Varying affinity exponent *a*

**Figure 4:**
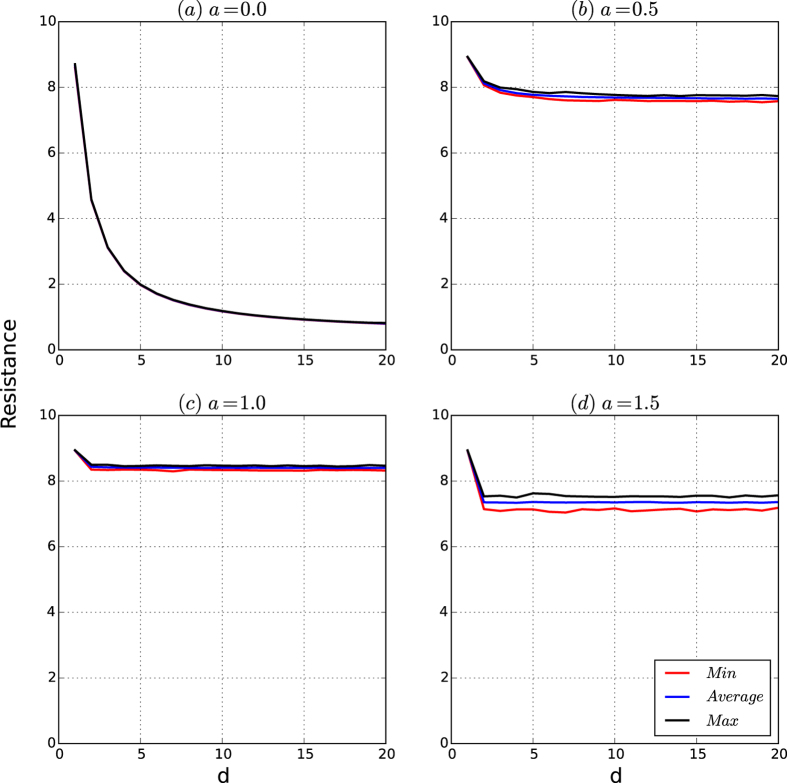
Resistances of the networks of the security model. The number of nodes is 10,000. For each type, we generate 200 networks. For each network, we approximate the resistance of the network based on the partition given by our resistance maximisation algorithm 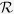. For each *a*, the minimum, average and maximum of the security resistances are taken over the 200 generated networks. (**a–d**) Are the curves of the resistances of the networks for *d* = 2, 4, 8 and 16, respectively.

Figure 4 depicts the resistances of the networks based on the resistance maximisation algorithm 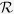.

From Fig. 4, we observe the following results. For each type, let *R*_avg_, *R*_min_ and *R*_max_ be the curves of the average, minimum and the maximum resistances of the 200 networks, respectively. Then:

1. (Robustness to affinity exponent *a* for small *a*) For the fixed *n*, the three curves *R*_avg_, *R*_min_ and *R*_max_ coincide within *a* ≤ *a*_0_ for some constant *a*_0_ ≈ 1 and branch for *a* > *a*_0_, for which the gaps among *R*_avg_, *R*_max_ and *R*_min_ increase as the affinity exponent *a* increases.
2. (Resistance is determined largely by the affinity exponent *a*) The resistance of the networks given by the communities found by resistance maximisation algorithm 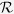 increases as the affinity exponent *a* increases up to some point *a*_0_ ≈ 0.8 and then decreases as *a* increases from *a*_0_.
3. (Strong resistance exists for an affinity exponent *a* that is not too small and not too large) The resistances of the networks given by the resistance maximisation algorithm 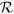 are high if the affinity exponent *a* is in some small interval (*a*_1_, *a*_2_) for some *a*_1_ and *a*_2_ with 0.5 < *a*_1_ and *a*_2_ < 1.5.

The results demonstrate that the robustness of the resistances of the networks of the security model is determined by the affinity exponent *a* and that for fixed *n*, there exists an interval (*a*_0_, *a*_1_) for the affinity exponent *a* such that for all *d*’s, the resistances of the networks are both robust to the random variations and invariant to varying *d*’s. However, the resistances of the networks of the security model are sensitive to the affinity exponent *a* when *a* is large. This result is not surprising because if *a* = 0, the networks of the security model are principally random graphs, whereas if *a* is large, there are only a few seed nodes in the networks, such that the networks are simply the union of a few large communities, each of which is a PA graph. According to this analysis, when the affinity exponent *a* increases, the networks of the security model change from uniformly random graphs to highly biased random graphs. Therefore, the important new properties of the security model can only be achieved for the affinity exponent *a* in some interval (*a*_0_, *a*_1_) in the case where the number *n* of the networks is given.

**Figure 5:**
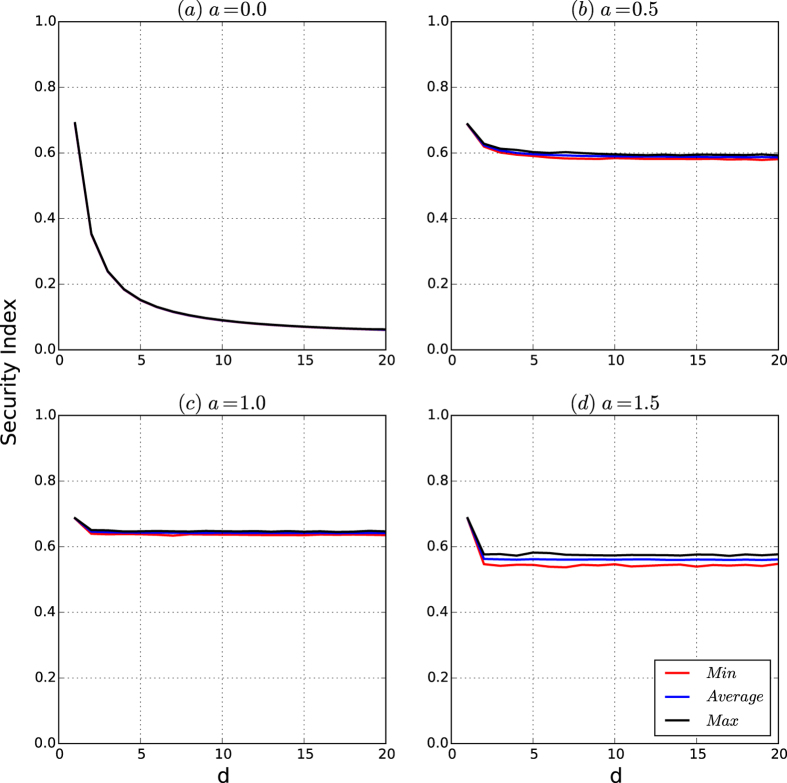
Security indices of the networks of the security model. The number of nodes is 10,000. For each type, we generate 200 networks. For each network, we approximate the security index of the network based on the partition given by our resistance maximisation algorithm 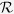. For each *a*, the minimum, average and maximum of the security indices are taken over the 200 generated networks. (**a–d**) Are the curves of the security indices of the networks for *d* = 2, 4, 8 and 16, respectively.

Figure 5 depicts the security index of the networks by the resistance maximisation algorithm 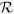. Figure 5 shows that the curves of the security indices of the networks of the security model are similar to that of the resistances of the networks shown in Fig. 4. Therefore, the security indices of the networks of the security model have the same properties as those for the resistances of the networks.

**Figure 6:**
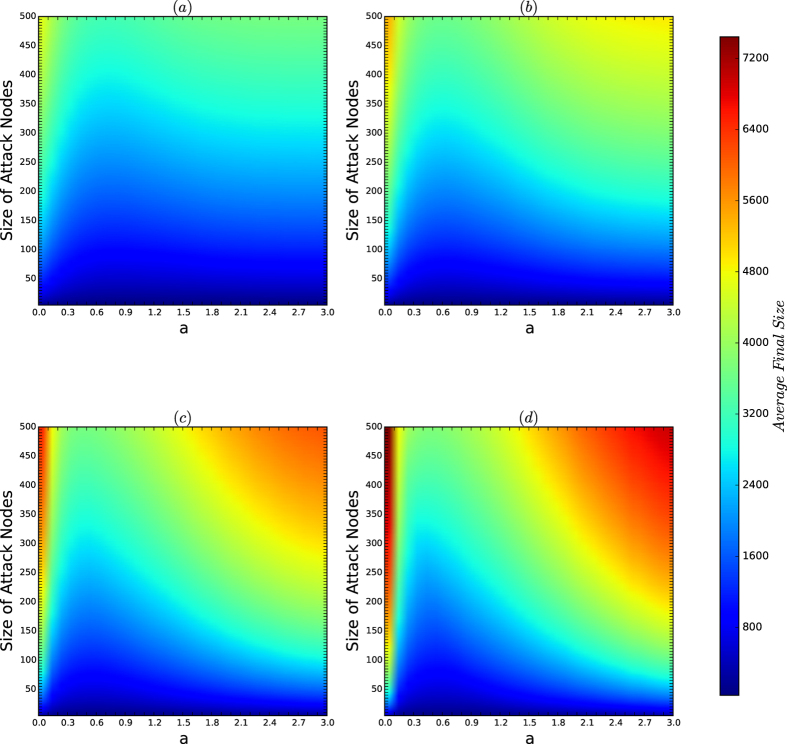
Colour codes of *I*_avg_ for the networks of the security model. In each experiment, *n* = 10,000, and *N* = *M* = 200. The affinity exponent *a* ranges from 0 to 3 with unit 0.1, and the size *k* of viruses ranges from 1 to 500 in steps of 1. The most influential *k* nodes are selected by the algorithm CLC, which is currently the best algorithm for finding the most influential nodes in networks. (**a–d**) Are the color codes of *I*_avg_ for the networks for *d* = 2, 4, 8 and 16, respectively.

Figure 6 depicts the colour codes for the average sizes of the infection sets of the attacks on the networks of the security model. In Fig. 6, we refer to the area that is coloured blue as the *secure area* in each of Fig. 6(a–d).

Figure 6 demonstrates the following results:

1. For *d* = 2, 4, the boundary of the secure area is similar to the curves of the resistances and the security indices in Figs 4 and 5, respectively. Thus, if *d* is small, for every *a*, the security of the networks of the security model is characterised by the resistance and the security index of the networks.
2. For *d* = 8, 16, if *a* is small, the secure area is measured by the resistance and the security indices in Figs 4 and 5, but if *a* is large, the secure areas in Fig. 6(c,d) are radically perturbed as *a* increases. The reason for the perturbation is as follows: for the fixed *n* = 10,000 used in our experiment, if both *a* and *d* are large, there are only a small number of (or a few) natural communities, the sets of all the nodes of the same colour, for each of the colours, and each of the natural communities is generated by the PA model with large *d*. Based on the experiments in Figs 1 and 2, we have shown that for a network of the PA model, the resistance and security indices and the secure areas of the networks exponentially decrease as *d* increases.
3. By (2), the security of the networks of the security model is determined by the resistance and security index with perturbation caused by large *a*’s and large *d*’s.

**Figure 7:**
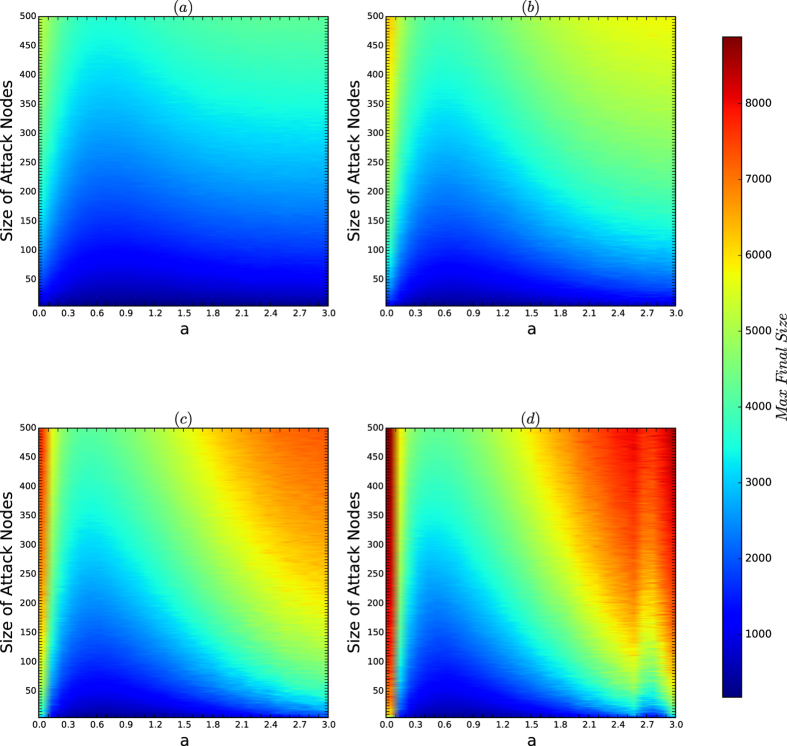
Colour codes of *I*_max_ for the networks of the security model. In each experiment, *n* = 10,000, and *N* = *M* = 200. The affinity exponent *a* ranges from 0 to 3 in steps of 0.1, the size *k* of viruses ranges from 1 to 500 with unit 1. The most influential *k* nodes are selected by the algorithm CLC, which is currently the best algorithm for finding the most influential nodes in networks. (**a–d**) Are the colour codes of *I*_max_ for the networks for *d* = 2, 4, 8 and 16, respectively.

Figure 7 depicts the colour codes of the maximum sizes of the infection sets of attacks on the networks of the security model.

By comparing Figs 6 and 7, we obtain the following results:

1. For each *a*, the security of the networks of the security model is determined by the resistances and security indices of the networks with perturbations.
2. The perturbation of the characterisation of the security by the resistances and security indices of the networks of the security model is determined by

- The sensitivity of the resistances and security indices of the networks, which occurs when the affinity exponent *a*’s are large, and
- The random variations in the same type of the model, which occurs for all types.

*Remarks:* we observe that in Fig. 7(d), when *a* = 2.7 and *d* = 16, the secure area is inconsistent with the resistances and security indices shown in Figs 4 and 5. Again the reason is the perturbation of the model when both *a* and *d* are large for a fixed *n*. In fact, when *a* is large, the resistance and security index are sensitive to random variations in the model, as explained in Figs 4 and 5.

In summary, we demonstrate the following results for the networks of the security model:

- If the affinity exponent *a* is not too large, both the resistances and the security indices of the networks are robust to random variations in the model.
- If the affinity exponent *a* is large, both the resistance and security index of the networks are sensitive to random variations in the model.
- In any case, the security of the networks of the security model is principally determined by the resistance and security index of the networks with perturbation that is caused by both the sensitivity of the resistance and security index and the random variations in the model.
- By appropriately choosing the affinity exponent *a* for fixed *n*, the resistances and security indices of the networks of the security model are both high and robust. Consequently, the networks of the security model with the corresponding types are guaranteed to be secure against any small number of virus attacks.

*Remark:* In real-world networks, there is no explicit parameter that corresponds to the affinity exponent *a* in our security model, although a real network may have some implicit affinity. Therefore, the role of affinity exponent *a* in real-world networks is implicit. The experiments for real-world networks, which are referred to in Table 1 and Fig. 14, show that the resistance and security index of the networks truthfully reflect the security of the networks against virus attacks of small size, regardless of whether the average degrees of the networks are small or large.

### Varying *d*

**Figure 8:**
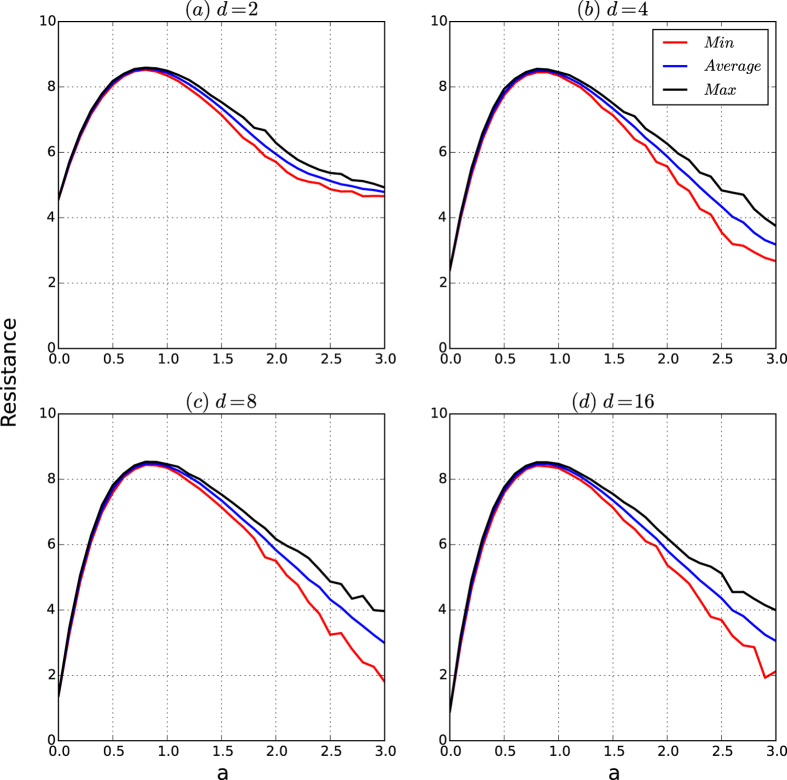
Resistance of the networks of the security model. The number of nodes is 10,000. For each type, we generated 200 networks of the model. The three curves in each of (**a–d**) are the maximum, average and minimum of the resistances of the 200 networks for each type, given by the partition found by the resistance maximisation algorithm 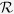. (**a–d**) Are for the networks with affinity exponents *a* = 0, 0.5, 1 and 1.5, respectively.

Figure 8 depicts the curves of the resistance of the networks of the security model as functions of *d*.

According to Fig. 8, we observe the following results:

1. (Robustness and exponentially decreasing property) Assume *a* = 0. According to Fig. 8(a), the maximum, average and minimum of the resistances of the networks of the security model are the same, and the resistance of the networks exponentially decreases as *d* increases. Thus, for *a* = 0, the networks of the security model are basically random graphs, in which case the resistances are robust to random variations in the model and exponentially decrease as *d* increases.
2. (Robustness and invariance) Consider a small affinity exponent *a*. According to Fig. 8(b,c), the maximum, average and minimum of the resistances of the networks are almost identical and are invariant for all *d* > 2.
3. (Sensitivity and invariance) Consider the case of large *a*. According to Fig. 8(d), the maximum, average and minimum of the resistances of the networks are slightly different, and the maximum, average and minimum of the resistances of the networks is invariant for *d* > 2.
4. If *a* is appropriately chosen, for all *d*, the resistances of the networks of the security model are both high and robust to random variations in the model.

**Figure 9:**
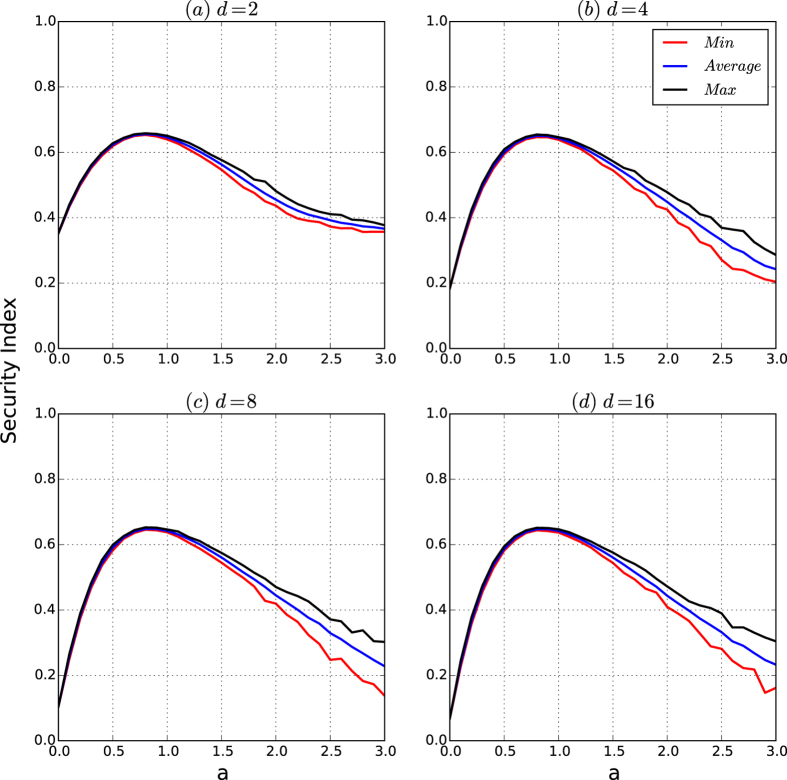
Security indices of the networks of the security model. The number of nodes is 10,000. For each type, we generated 200 networks of the model. The three curves in each of (**a–d**) are the maximum, average and minimum of the security indices of the 200 networks for each type, given by the partition found by the resistance maximisation algorithm 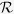. (**a–d**) Are for the networks with affinity exponents *a* = 0, 0.5, 1 and 1.5, respectively.

Figure 9 depicts the security indices of the networks of the security model as *d* increases. Figure 9 shows that the curves of the security indices of the networks of the security model are the same as those for the resistances of the networks in Fig. 8.

**Figure 10:**
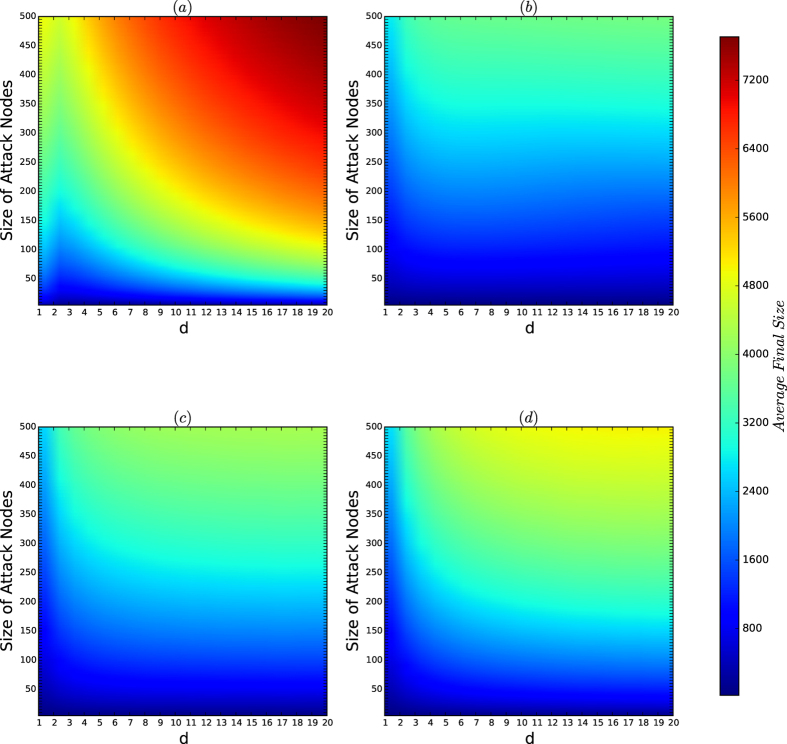
Colour codes of the average cascading failure sets of virus attacks on the networks of the security model. The number of nodes is 10,000. *N* = *M* = 200. (**a–d**) Are for the networks with affinity exponents *a* = 0, 0.5, 1 and 1.5, respectively.

**Figure 11:**
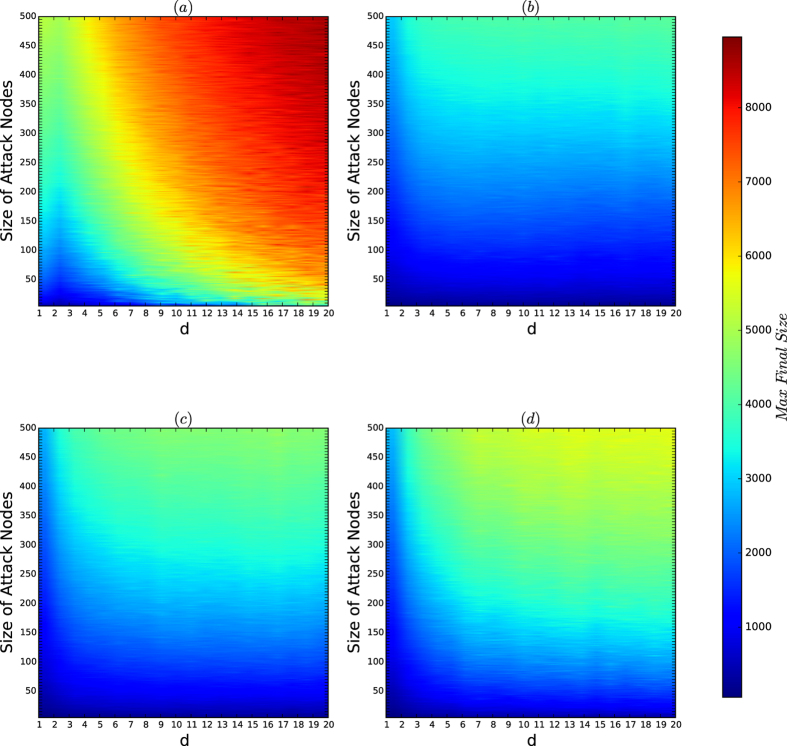
Colour codes of the maximal cascading failure sets of virus attacks on the networks of the security model. The number of nodes is 10,000. *N* = *M* = 200. (**a–d**) Are for the networks with affinity exponents *a* = 0, 0.5, 1 and 1.5, respectively.

Figure 10 depicts the colour codes of the average sizes of the cascading failure sets of attacks on the networks of the security model as *d* increases. Figure 11 depicts the colour codes of the maximum sizes of the cascading failure sets of attacks on the networks of the security model as *d* varies.

According to Figs 10 and 11, we observe that for each choice of *a*, the secure areas, i.e., the areas coloured blue, are principally determined by the resistances and security indices of the networks with a slight perturbation caused by random variations in the model because the affinity exponent *a* in this experiment is ≤1.5, which is not large.

According to Figs 8, 9, 10, 11, we have that for a fixed *n* (10,000 in our experiments), if the affinity exponent *a* > 0 is not too large, then the following hold:

1. The resistance of the networks of the security model is both robust to random variations and invariant as *d* changes beyond 2.
2. The security index of the networks of the security model is both robust and invariant as *d* changes beyond 2.
3. The security of the networks of the security model is principally determined by the resistance and security index of the networks with slight perturbations caused by random variations in the model.

The results demonstrate that for any number of nodes *n* and for any density parameter *d*, there exists an interval (*a*_0_, *a*_1_) for the affinity exponent such that the networks of the security model have high and robust resistances and security indices, which guarantee the security of the networks against cascading failure from any small-scale virus attacks.

### Varying *a* and *d*

**Figure 12:**
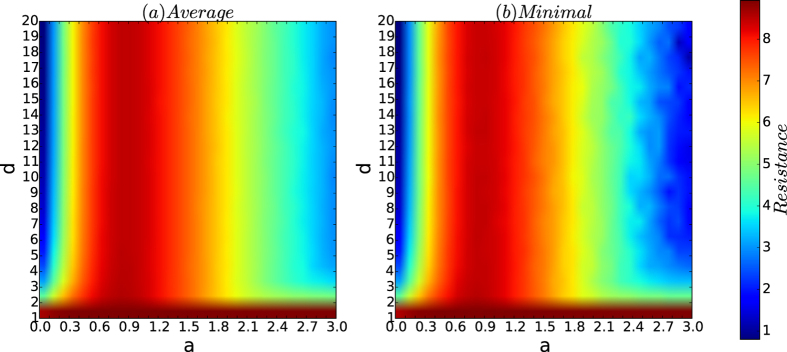
The colour codes of the resistance of the networks of the security model as *a* and *d* vary. The number of nodes is 10,000. For each type, we generate 200 networks. (**a,b**) Are the colour codes of the average and minimal resistances of the networks. The resistance of a network is given by the partition found by the resistance maximisation algorithm 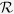.

**Figure 13:**
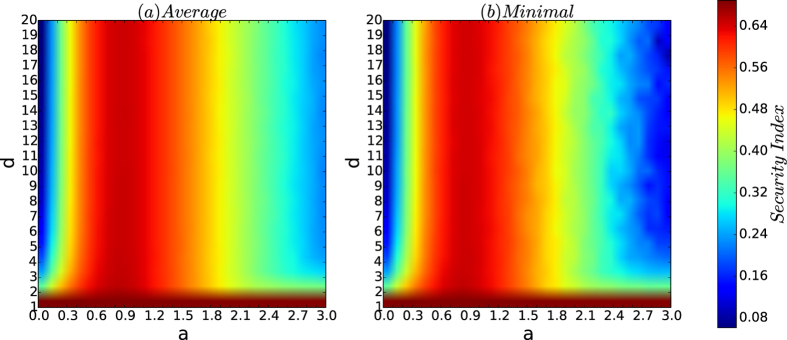
The colour codes of the security index of the networks of the security model as *a* and *d* vary. The number of nodes is 10,000. For each type, the security indices of the networks are computed as the average and minimum of the security indices over 200 networks. (**a**,**b**) Are the colour codes of the average and minimum of the security indices of the networks over the 200 attacks for each of the 200 networks, for each type. The security index of a network is given by the partition found by the resistance maximisation algorithm 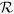.

Figure 12 depicts the resistance of the networks of the security model as *a* and *d* vary. Figure 13 depicts the colour codes of the security index of the networks of the security model as *a* and *d* vary.

From Figs 12 and 13, we observe the following results:

1. If *d* = 1, for any affinity exponent *a*, both the resistances and security indices of the networks are robust and high.
2. For both the average and minimum of the resistances and the security indices, for every fixed affinity exponent, the colour belts of both resistances and security indices have the same or almost the same colour as *d* varies.
3. In each of the Figs 12(a,b) and 13(a,b), the deepest red area is roughly a rectangle for *a* ∈ (0.5, 1) for all *d*’s.

*Remark:* (i) There is a trivial solution for the construction of networks to minimise the one-, and two-dimensional structure entropy; we simply take the isolated nodes without any edges. (ii) Maximisation of the one-dimensional structure entropy requires creating the maximum amount of uncertainty in the random walks in *G*. Therefore, resistance maximisation is a well-defined problem for constructing networks. However, structure entropy minimisation alone is not a well-defined problem for constructing networks because the problem itself has a trivial solution. In network engineering, it would be better to use the resistance maximisation principle. In noisy data analysis, it is better to use the structure entropy minimisation principle.

Figures 12 and 13 demonstrate that for a fixed number *n* of nodes, there is a large interval (0.3, 1.5) such that for every *d*, and for every affinity exponent *a* in the interval, the resistances and the security indices of the networks of the security model are both high and robust, thus ensuring that the corresponding networks are guaranteed to be secure against cascading failures from any small-scale virus attacks.

## Resistance and Security Indices of Real-World Networks

We examine five real-world networks, the Blog, Yeast, OpenFlights, the US power grid and a co-author graph. Details can be found in the Methods section.

Table 1 describes the resistances and security indices of four real world networks. The average degrees of the graphs ranging from 2.669 to 64.776.

**Figure 14:**
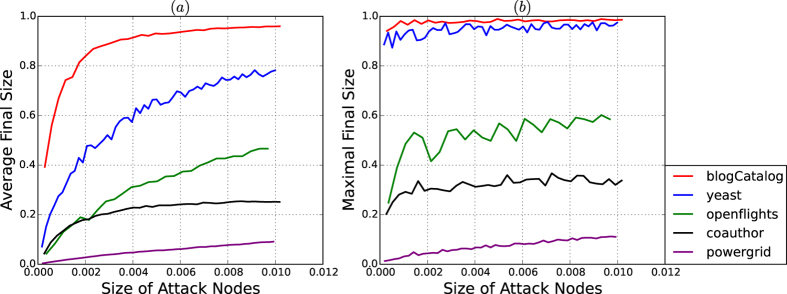
Security of real-world networks. For each network, we implemented 200 attacks. For each attack, we picked the thresholds for all the nodes randomly. The size of the attack is the fraction of the nodes of the network. The targeted nodes were selected by the CLC algorithm. (**a,b**) Are the average and maximum of the cascading failure, taken over the 200 attacks for each size.

Figures 14(a,b) depict the average and maximum sizes of the cascading failures from attacks on the five real-world networks.

The experiment here is as follows: for each size *k* of viruses, we implement 200 attacks. For each attack, we define the threshold for each node as a random number, and infect the most influential *k* nodes by the *k* viruses. The average and maximum sizes of the cascading failure sets are computed over the 200 attacks for each size *k* of virus.

According to Table 1 and Fig. 14, we observe that the curves of both the average and maximum fractions of the cascading failure sets of the small-scale attacks are consistent with the resistances and security indices of the networks.

We remark that the result above holds for the networks of both small and large average degrees, meaning that the perturbation caused by large *a* and large *d* (with fixed *n*) for the networks of the security model does not occur in real-world networks.

The experiments show that for each real-world network, the security of the network against cascading failure caused by any small-scale virus attack is fully reflected in both the resistance and the security index of the network.

## Conclusions and Discussions

We proposed the notions of resistance and the security index of networks. We found both the local and global resistance laws of networks. We proposed an algorithm on the basis of resistance maximisation to approximately compute the resistance and the security index of networks. We investigated the resistance, the security index and the security of the networks generated by the preferential attachment model and the security model. We also investigated the resistance, the security index and the security of real-world networks.

Our theory shows the following:

- For a model 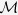, the security of the networks of the model is characterised by the resistance and security index of the networks with perturbation by random variations in the model 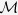.
- For the PA model, both the resistance and security index of the networks are robust to random variations in the model and are exponentially decreasing as *d* increases.
- For the security model, there is an interval (*a*_0_, *a*_1_) for the affinity exponent such that both the resistance and security index of the networks of the model are high, robust to random variations, and invariant to *d*, which ensures that the corresponding networks are guaranteed to be secure against any small-scale virus attacks. Therefore, secure networks of various sizes with various connectivity requirements *d* are guaranteed by the security model with appropriate choices of the affinity exponent. Furthermore, the resistance and security index provide the criteria to choose the optimal affinity exponent *a* for constructing the best possible networks for the security model; this approach is useful for engineering applications.
- For the security model with a fixed affinity exponent *a*, the resistances and security indices of the networks are invariant to *d* > 1.
- For a model 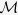 of networks, the security of the networks of the model is always sensitive to the random variations of the model 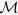, in the sense that, networks of the same type generated by model 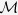 may have different security performances. However, the resistances and security indices of the networks of the model are robust to random variations in the model. Therefore, there is always a perturbation for the characterisation of the security of the networks of the model based on the resistances and the security indices. Our results demonstrate that for the PA model and the security model with appropriately small affinity exponent *a*, the perturbation of the characterisation is small, so that the resistance and security index are both well-defined metrics for characterising the security of the networks of the PA and security models. It is reasonable to believe that the same result holds for the other models.
- For the security model 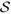 with bounded size *n*_0_, the resistances and the security indices of the networks of the model with very large affinity exponent *a* are sensitive to both the varying of *a*’s and random variations in the model. This sensitivity indicates that the perturbation of the characterisation of the security by the resistances and security indices of the networks of the security model with large affinity exponent is large; if *a* is large, a network of the security model is simply the union of a few PA graphs and thus loses the essential properties of the networks of the security model.
- For real-world networks, we show that the security of the networks is perfectly characterised by the resistances and security indices of the networks. This result further ensures that for a given network *G*, the resistance and the security index of *G* are both well-defined metrics for the security of *G* against intentional virus attacks of small size.

The results have important implications for communication networks, computer networks and networking systems of computation. For instance, we now know that the notions of resistance and a security index are well-defined metrics for the security of engineering networks and that resistance maximisation or security index maximisation is one of the principles of engineering networks.

Our theory suggests the following theoretical directions for communication networks: 1) to investigate the relationships among the engineering requirements of engineering networks such as congestion minimisation, expansion maximisation and the resistance maximisation principle and 2) to show that the optimal systems for communications require that the engineering requirements and the resistance maximisation principle are all satisfied, for which a hierarchical structure of the systems is necessary. The hierarchical structure is necessary because it seems impossible to satisfy all the engineering requirements and the resistance maximisation principle in a single level of the systems. Nevertheless, there is a method to satisfy the engineering requirements and the resistance maximisation principle at different levels of the systems. For example, expansion maximisation requirement may conflict with the resistance maximisation principle because the former requires the spread of information to be immediate, and the latter requires the spread of viruses to be difficult. Our theory suggests a solution to this problem as follows: the network consists of many small modules; the connections among the modules are expanders with good expansion properties, and in each module, there is a base (seed) node that plays the role of a “guard” (checking virus) for its own module (which is small). Research in this direction may establish a theoretical foundation for the engineering of networks.

Our results suggest a new theoretical approach to network science. It is interesting to establish theoretical results regarding the robustness and sensitivity of the resistances and security indices of the networks for various models.

Our results show that for appropriate choices of the affinity exponent *a*, the resistances and security indices of the networks generated by the security model are high. However, the security model uses randomness as one of its mechanisms. A new challenge is to give a deterministic polynomial time algorithm to construct networks of *n* nodes with an average number of edges *d* such that the resistance or the security index of the network is maximised. Generally, we say that a graph *G* is an (*n*, *d*, *ρ*)-*resistor graph*, if *G* has *n* nodes and an average number of edges *d* such that the security index of *G* is at least *ρ*. It is interesting to design deterministic polynomial time algorithms to construct an (*n*, *d*, *ρ*)-resistor graph for large *ρ*, for all *n* and *d*. The resistor graphs may be devices for engineering networks. Clearly, this question is fundamental to many applications of networks. To better understand this, let us examine some examples. The first example is cloud computing; it is possible that resistor graphs are good models for cloud computing because in a resistor graph, most interactions are within small modules and a small number of edges create the expansion property of the whole graph in a secure manner. The second example is that intuitively, a local search is extremely easy and fast in a resistor graph; from every node of the graph, we may immediately identify the natural module of the node. This example implies that the idea of resistor graphs may be used to develop new principles for distributed computing.

Additional topics left unaddressed by this research include the following: 1) new algorithms for the resistance maximisation problem; 2) investigating the problem of network control based on the resistance theory; 3) developing other characterisations of the resistance and security index of graphs, including combinatorial characterisation and algebraic characterisation based on eigenvalues. Research regarding these topics is important for both information science and computer science.

Finally, we note that the security of networks is the security against cascading failure from virus attacks. The immediate questions include the following: do the resistance and security index measure the security of networks against physical attacks of removal of nodes and edges and the biological virus in the SIR model^28^? Intuitively, the answer to this question is yes. Suppose that *G* is a network with high resistance 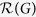. Then, there is a partition 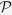 of vertices of *G* consisting of small modules among which random walks are hard to cross over. In this case, for the physical attack model, deleting a small number of nodes may only disconnect a small number of small modules from the remaining giant connected component. For the SIR model, a single biological virus randomly spreads in *G* with a mechanism of recovery with some probability. The situation is very similar to the cascading model. In both cases, the high resistance of a network suggests the strength of the security of the network. However, development of the theory requires a separate study.

## Methods

### Resistance maximisation algorithm 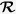


According to the resistance law, 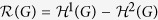. We also notice that it is difficult to precisely compute the resistance of *G* because it represents the maximum values overall the partitions of *G*. Therefore, we can only compute an approximate solution for the resistance maximisation of *G*. In addition, for a given graph *G*, the one-dimensional structure entropy 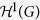 of *G* is fixed by the distribution of degrees of *G*. Therefore, maximising the resistance of *G* is equivalent to minimising the two-dimensional structure entropy of *G*. We design our resistance maximisation algorithm 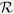 by minimising the two-dimensional structure entropy of *G*.

We will use a simple greedy algorithm to find a partition that minimizes the two-dimensional structure entropy of the network *G* introduced in Li, Li and Pan^24^ and Li *et al*.^25^.

Suppose that 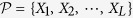 is a partition of *V*. For *i*, *j* with 1 ≤ *i*, *j* ≤ *L*, by the definition given in Equation (2) , if we obtain a partition 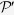 from 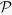 by merging *X*_*i*_ and *X*_*j*_, the difference of the structure entropies given by the two partitions is given by


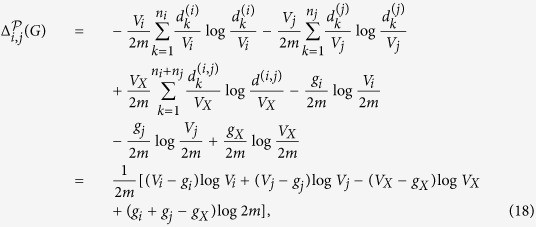


where 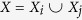, *V*_*X*_ is the volume of *X*, *g*_*X*_ is the number of edges from *X* to nodes outside of *X*, 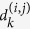 is the degree of the *k*-th node in *X*.

If there is no edge between *X*_*i*_ and *X*_*j*_, then *g*_*X*_ = *g*_*i*_ + *g*_*j*_. In this case,


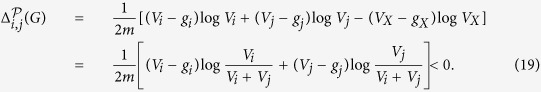


Therefore, 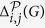 is locally computable, and if there is no edge between *X*_*i*_ and *X*_*j*_, 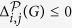.

The resistance maximisation algorithm, written as 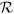, proceeds as follows.

Given a network *G*:

1. Set the initial partition such that each module contains a single node.
2. Recursively merge the modules *X*_*i*_ and *X*_*j*_ such that the corresponding 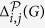 is maximized, until there is no such merging operation, in which case, output the corresponding partition 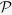.

It has been shown that the algorithm 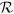 exactly identifies or precisely approximates true communities in many networks generated by models or in real-world networks^24^,^25^,^26^.

### Resistances and security indices

For a model 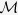, for each type, we generate *N* networks *G*_1_, *G*_2_, ···, *G*_*N*_, for each *i* ∈ {1, 2, ···, *N*}, we compute the resistance and security index of *G*_*i*_ according to the resistance maximisation algorithm 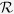.

The minimum, average and maximum of the resistances and security indices are taken over all of the *N* networks.

For the models in our experiments, we always choose *n* = 10,000 and *N* = 200.

### Cascading failure from virus attacks

Let *G* = (*V*, *E*) be a network. Suppose that for every node *v* ∈ *V*, there is a threshold *ϕ*_*v*_ ∈ (0, 1]. Given a set *S* of nodes, define a set *I* as follows:

- every *x* ∈ *S* is in *I*, and
- for every node 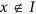, if *ϕ*_*x*_ fraction of neighbours of *x* is in *I*, then *x* enters *I*.

We say that *I* is the *infection set of S in G with respect to threshold ϕ*, denoted by 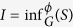.

It is interesting to note that the definition of the infection set depends on a threshold function *ϕ*_*v*_ for all node *v*’s in *V*. The threshold function *ϕ* maybe an arbitrary distribution. However, the purpose of our study is to investigate the role of structures of *G* in the security of networks. For this purpose, we assume that the threshold *ϕ* is randomly and uniformly distributed. Thus, for every node *v*, we define 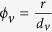, where *d*_*v*_ is the degree of *v* in *G*, and *r* is randomly and uniformly picked from {1, 2, ···, *d*_*v*_}. Throughout the study, we always assume the random definition of the threshold function *ϕ*.

Given *G* = (*V*, *E*) with a random threshold *ϕ*, we characterise the security of network *G* as follows: with high probability, for any small set *S* ⊂ *V*, the infection set *I* of *S* in *G* with *ϕ* is small, i.e., for any set *S*, if *S* is small, so is 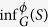.

### CLC strategy of attacks

We will use the algorithm by Moores *et al*.^29^ to find the sets of the nodes to attack. The algorithm is based on the notion of combinatorial local centrality and is denoted by CLC. The algorithm finds the most influential set of *k* nodes with approximation ratio 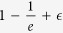. It is slightly better than even the algorithm given by Kempe, Kleinberg and Tardos^7^.

To describe our results, we define some notations.

A type of attack for a model of networks proceeds as follows:

Let *n* = 10,000.

For each type of the model, and each size *k* of attacks, we generate *N* networks, *G*_1_, *G*_2_, ···, *G*_*N*_. For each *i* from 1 to *N*, we define *M* distributions *ϕ*_*i*,1_,*ϕ*_*i*,2_, ···,*ϕ*_*i*,*M*_ such that *ϕ*_*i*,*j*_ is a randomly defined threshold function of network *G*_*i*_.

For every pair (*i*, *j*), for 1 ≤ *i* ≤ *N* and 1 ≤ *j* ≤ *M*, and for every *k*, let 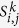 be the set of the most influential *k* nodes of network *G*_*i*_ with threshold function *ϕ*_*i*,*j*_ found by the algorithm CLC, which are approximated the most influential *k* nodes in the network *G*_*i*_.

We define


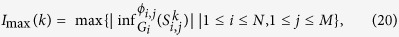



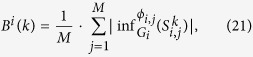



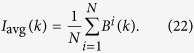


Therefore, *I*_max_(*k*) is the largest size of the infection sets among *M* attacks on the most influential *k* nodes found by the algorithm CLC for each of the *N* networks of the given type of the model.

*I*_avg_(*k*) is the average size of the infection sets among *M* attacks on the most influential *k* nodes for each of the *N* networks of the given type of the model.

We investigate both *I*_max_ and *I*_avg_ as functions of the size *k* of viruses of the attack.

In all the experiments for the networks of models, for each type, we choose *n* = 10,000 and implement the experiments with *N* = *M* = 200.

For real world networks, the graphs are fixed. In this case, the experiments are implemented by using *M* = 200 for each size *k* of viruses.

### Real world networks

The five real networks are as follows:

1. Blog Number of nodes: 10312, number of edges: 333983. This is the data set crawled from BlogCatalog (http://www.blogcatalog.com). BlogCatalog is the social blog directory which manages the bloggers and their blogs. Both the contact network and selected group membership information are included. The data can be found in [http://socialcomputing.asu.edu/datasets/BlogCatalog3].
2. Yeast It contains 2224 nodes, and 6609 edges. Protein-protein interaction network in budding yeast. The data can be found in [http://vlado.fmf.uni-lj.si/pub/networks/data/bio/Yeast/Yeast.htm].
3. Openflights
4. It contains 2790 nodes and 14795 edegs. This directed network contains flights between airports of the world. A directed edge represents there is a flight between two airports. This dataset is extracted from Openflights.org data and corresponds to network 14c in the dataset list by Tore Opsahl. The data can be found in [http://konect.uni-koblenz.de/networks/opsahl-openflights].
5. Powergrid It contains 4941 nodes and 6594 edges. This undirected network contains information about the power grid of the Western States of the United States of America. An edge represents a power supply line. A node is either a generator, a transformator or a substation. The data can be found in [http://konect.uni-koblenz.de/networks/opsahl-powergrid].
6. Coauthor The graph contains 11204 nodes and 117619 edges. Arxiv HEP-PH (High Energy Physics - Phenomenology) collaboration network is from the e-print arXiv and covers scientific collaborations between authors papers submitted to High Energy Physics - Phenomenology category. The data can be found in [http://snap.stanford.edu/data/ca-HepPh.html].

## Additional Information

**How to cite this article**: Li, A. *et al*. Resistance and Security Index of Networks: Structural Information. Perspective of Network Security. *Sci. Rep.* **6**, 26810; doi: 10.1038/srep26810 (2016).

## Figures and Tables

**Table 1:** Resistance and security index of four real world networks.

Networks	Resistance	Security Index	Average degree
Blog	0.647	0.055	64.776
Yeast	1.085	0.090	5.943
OpenFlights	2.531	0.254	10.606
PowerGrid	6.996	0.583	2.699
Coauthor	3.467	0.293	20.995
